# Impact of breastfeeding and other early-life factors on the development of the oral microbiome

**DOI:** 10.3389/fmicb.2023.1236601

**Published:** 2023-09-07

**Authors:** Roaa A. Arishi, Ching T. Lai, Donna T. Geddes, Lisa F. Stinson

**Affiliations:** ^1^School of Molecular Sciences, The University of Western Australia, Perth, WA, Australia; ^2^Ministry of Health, Riyadh, Saudi Arabia

**Keywords:** oral microbiome, infant diet, breastfeeding, HMOs, lactoferrin, lysozyme, sIgA

## Abstract

The oral cavity is home to the second most diverse microbiome in the human body. This community contributes to both oral and systemic health. Acquisition and development of the oral microbiome is a dynamic process that occurs over early life; however, data regarding longitudinal assembly of the infant oral microbiome is scarce. While numerous factors have been associated with the composition of the infant oral microbiome, early feeding practices (breastfeeding and the introduction of solids) appear to be the strongest determinants of the infant oral microbiome. In the present review, we draw together data on the maternal, infant, and environmental factors linked to the composition of the infant oral microbiome, with a focus on early nutrition. Given evidence that breastfeeding powerfully shapes the infant oral microbiome, the review explores potential mechanisms through which human milk components, including microbes, metabolites, oligosaccharides, and antimicrobial proteins, may interact with and shape the infant oral microbiome. Infancy is a unique period for the oral microbiome. By enhancing our understanding of oral microbiome assembly in early life, we may better support both oral and systemic health throughout the lifespan.

## 1. Introduction

The oral microbiota inhabit and interact with the oral cavity, playing an essential role in oral health (Jia et al., 2018; Nardi et al., 2021). As such, perturbations to the oral microbiome, have been associated with various conditions, including dental caries, periodontal diseases, and pericoronitis (Colombo and Tanner, 2019). Alterations to the oral microbiome have been also been associated with systemic disease, including diabetes mellitus (Chen et al., 2020; Matsha et al., 2020; Qin et al., 2022), atherosclerotic disease (Ford et al., 2006), inflammatory bowel disease (Ismail et al., 2012), respiratory infections (Heo et al., 2008), and rheumatoid arthritis (Martinez-Martinez et al., 2009; Dissick et al., 2010). Consequently, the oral microbiome has been considered a potential biomarker for local and systemic health.

World Health Organization (WHO) (2021a) recommends human milk as the exclusive food source for infants for the first 6 months of life. Human milk promotes health by providing infants with nutritive and bioactive components (including microbes, metabolites, oligosaccharides, and antimicrobial proteins). Given increasing evidence demonstrating that the microbial and non-microbial components of human milk shape the infant gut microbiome (Lewis et al., 2015; Asnicar et al., 2017; Ho et al., 2018; Ma et al., 2020), it is highly likely that they also influence early oral microbiome dynamics. Indeed, the mouth is the obligatory transition point for milk to enter the gastrointestinal tract. Thus, the influence of infant diet on the development of the oral microbiome should be considered. Such studies are crucial in light of the unresolved question on whether prolonged breastfeeding duration is causative of dental caries (Iida et al., 2007; Nobile et al., 2014; Chaffee et al., 2015; Devenish et al., 2020; Carrillo-Diaz et al., 2021), necessitating high quality data to inform feeding decisions for parents.

The aim of this review is to describe oral microbiome development in early life, with a particular focus on the impact of breastfeeding and infant diet. We will review the evidence for the determinants of the early oral microbiome more broadly and will highlight potential mechanisms through which breastfeeding may impact the oral microbiome. Improving our understanding of the infant oral microbiome will contribute to evidence-based support for promoting and maintaining both oral and systemic health.

## 2. Methods

A search was performed in PubMed, Web of science, Scopus, Google Scholar, ProQuest and One Search (English) with the following combinations of search terms: “infant/ toddler/early life /temporal development” with “oral / saliva / buccal/salivary/plaque / mouth” and “microbiome/microbiota/bacteria.” The search covers the prior to August 2023.

## 3. Composition and temporal development of the infant oral microbiome

Early-life is a curical window of time for establishment and development of the oral microbiome. To date, there are relatively few studies examining temporal development of the oral microbiome, and those that do exist largely include low numbers of participants (< 300). Compared to the infant gut microbiome, the composition and development of the oral microbiome has received little attention, resulting in a poor understanding of this important environment. Current evidence suggests that similar to the adult oral microbiome, the infant oral microbiome is dominated by Streptococcus (Drell et al., 2017; Verma et al., 2018). It is also comprised largely of facultative anaerobes such as Gemella, Rothia, Haemophilus, and Lactobacillus (Blum et al., 2022). After birth, the neonatal oral microbiome is dominated by *Streptococcaceae, Staphylococcaceae, Gemellaceae, Pasteurellaceae,* and *Lactobacillales* (Gomez-Arango et al., 2017; Dashper et al., 2019; Sulyanto et al., 2019; Williams et al., 2019; Holgerson et al., 2020; Cheema et al., 2022). The oral microbiome is particularly unstable at this early stage, with some early colonizers such as *Staphylococcus epidermidis*, which makes up 11.1% of the bacterial profile at 2–5 days, disappearing over time (<1% relative abundance by 30 days) (Cheema et al., 2022).

Infancy is a dynamic period for the oral microbiome, marked by the emergence of teeth, mouthing behaviors, and the introduction of solid foods. Within the first 3 months of life, the oral cavity harbors a simple core microbiome made up of Streptococcus, Veillonella, Gemella, and Rothia (Nelson-Filho et al., 2013; Mason et al., 2018; Dashper et al., 2019; Sulyanto et al., 2019; Ramadugu et al., 2021). Genera such as Neisseria, Gemella, Granulicatella, Veillonella, Haemophilus, and Prevotella have been reported to increase at 3–6 months (Cephas et al., 2011; Dzidic et al., 2018). With age, the infant oral microbiome increases in complexity and richness (Sulyanto et al., 2019; Ramadugu et al., 2021; Li et al., 2023).

The trend of increased microbiome diversity continues into early toddlerhood, before stabilizing in early childhood (Dzidic et al., 2018; Dashper et al., 2019; Holgerson et al., 2020; Kahharova et al., 2020). In one study of 119 toddlers aged 1 to 4 years, significant alterations were identified in the microbial composition of both saliva and dental plaque over time (Kahharova et al., 2020). Diversity increased from 1 to 2.5 years but remained stable from 2.5 to 4 years. Although similarity between toddler and care-giver oral microbiomes increased over time, the oral microbiome at 4 years of age was not as complex as that of their caregivers at this time. Similarly, in a study of 134 children and their mothers, alpha diversity of the infant oral microbiome remained stable over 3–5 years of age, with the oral microbiome of children at 5 years of age still significantly distinct from that of their mothers in terms of diversity and composition (Dashper et al., 2019). This suggests that maturation into an adult-like microbiome occurs in later childhood or beyond. The dominant bacterial genera at 1–8 years of age have been reported to be *Enterobacteriaceae*, Streptococcus, Gemella, Veillonella, Haemophilus, Prevotella, Granulicatella, Capnocytophaga, Leptotrichia, Actinomyces, Corynebacterium, Fusobacterium, Porphyromonas, and Neisseria (Xin et al., 2013; Dzidic et al., 2018; Li F. et al., 2018; Dashper et al., 2019; Holgerson et al., 2020; Kahharova et al., 2020). Streptococcus, which is highly abundant in the early oral microbiome, gradually declines in relative abundance across the first 7 years of life, accompanied by a corresponding increase in Gemella, Granulicatella, Haemophilus, Rothia, Actinomyces, Porphyromonas, and Neisseria (Dzidic et al., 2018).

**Figure 1:**
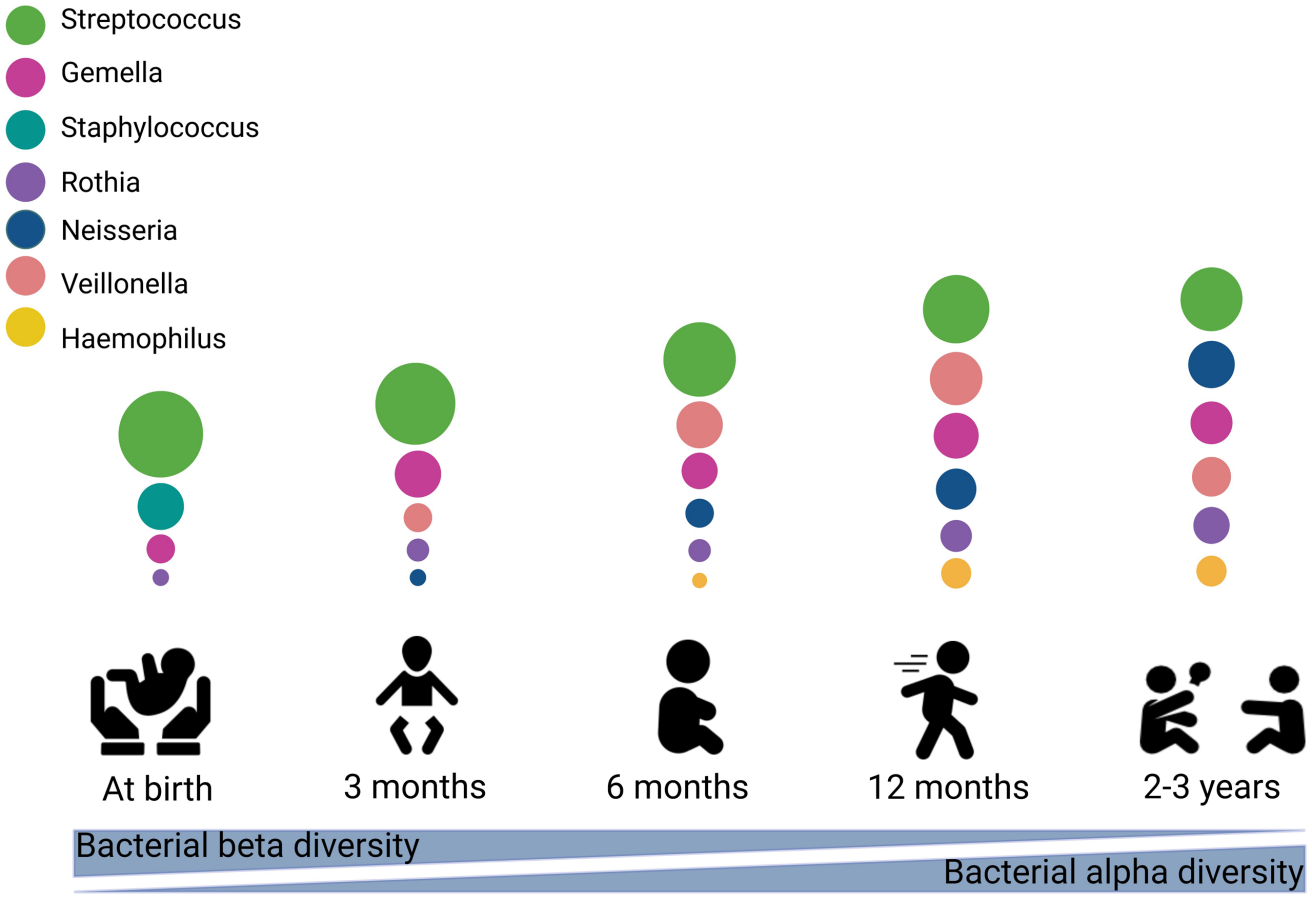
Stages of microbial colonization of the oral cavity. The most abundant bacteria are depicted in circles, where the size of the circle is proportional to the relative abundance of the bacterial taxa at each growth stage. This figure created with BioRender.com.

**Figure 2:**
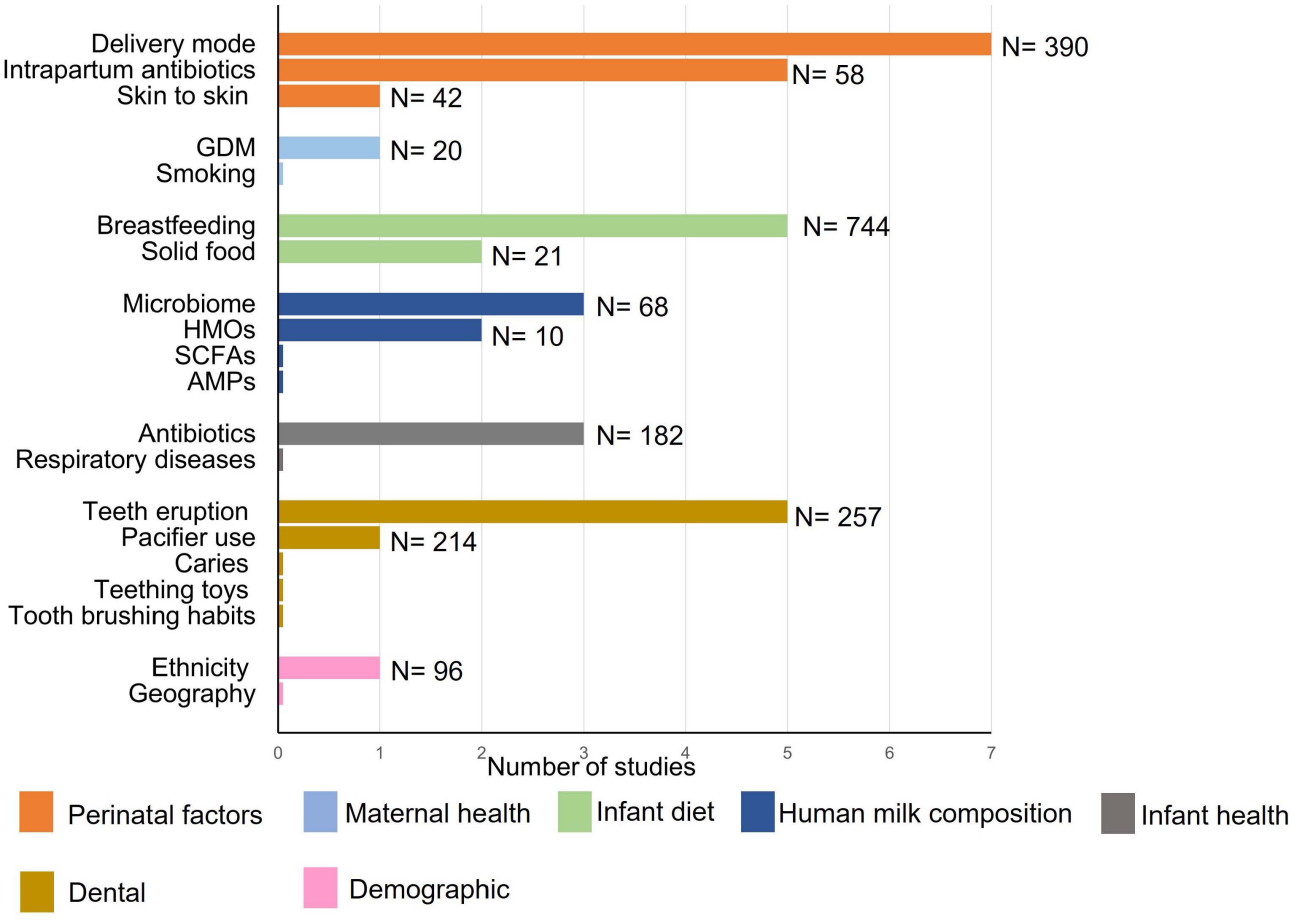
Numbers of publications with sample sizes exploring associations between maternal, infant, and environmental factors, and the infant oral microbiome. DM, delivery mode; IAP, intrapartum antibiotic prophylaxis; GDM, gestational diabetes mellitus; HMOs, human milk oligosaccharides; SCFA, short chain fatty acids; AMPs, antimicrobial proteins.

Collectively, current evidence suggests that the early oral cavity is dominated by *Streptococcus* spp., which may disappear gradually with age. A rapid increase in in alpha diversity and a decrease in beta diversity has been observed (Kennedy et al., 2019), similar to observations reported within the infant gut microbiome (Moore and Townsend, 2019; Cheema et al., 2022). Current studies suggest that acquisition and establishment of the oral microbiome is a dynamic process (Figure 1); however, very few studies have assessed associations between relevant maternal, infant, and environmental factors and the oral microbiome (Figure 2). Thus, it is important to understand the origin, composition, and timing of colonization of the infant oral microbiome, and how these microbial communities evolve over the early stages of life.

## 4. Diet

### 4.1. Effect of human milk vs. formula feeding on the infant oral microbiome

Breastfeeding is a major determinant of the infant oral microbiome (Al-Shehri et al., 2016; Oba et al., 2020; Butler et al., 2022). The difference between breast-and formula-fed infants begins within days of birth, with the oral cavity of exclusively breastfed neonates 24–48 h post-partum exhibiting a higher abundance of *Actinobacillus* and *Neisseria* and a lower abundance of *Haemophilus* than those who received both breast milk and infant formula (mixed-fed) (Oba et al., 2020). At two months of age, breastfed infants harbor a higher abundance of Streptococcus and lower abundance of Veillonella (Oba et al., 2020; Butler et al., 2022), and at three months of age, lactobacilli are only detected in exclusively and partially breastfed infants (Holgerson et al., 2013). At six months of age a lower abundance of *Weeksellaceae* is seen compared with mixed-fed infants (Oba et al., 2020). At the phylum level, a higher abundance of Bacteroidetes and a lower abundance of Proteobacteria are observed in formula-fed infants (Al-Shehri et al., 2016; Butler et al., 2022). Studies comparing breast-and formula-fed infants consistently find a lower alpha diversity in the oral cavity of breastfed infants (Al-Shehri et al., 2016; Williams et al., 2019; Oba et al., 2020; Butler et al., 2022), consistent with the effect of breastfeeding on the infant gut microbiome (Ho et al., 2018).

### 4.2. Mechanisms by which human milk may shape the oral microbiome

Human milk is comprised of various microbial, bioactive, and nutritional components, which may influence the oral microbiome. However, direct evidence regarding these interactions is lacking. Studies to address this gap include cohort studies linking milk composition to oral microbiome composition, whole genome sequencing studies to identify metabolic genes of interest in the infant oral microbiome that may interact with various milk components, and culture supplementation studies to examine the impact of various milk components on cultured infant oral taxa. Potential mechanisms of action are discussed below.

#### 4.2.1. Human milk bacteria may colonize the infant oral cavity

Human milk contains 10^6^ bacterial cells/mL (Boix-Amorós et al., 2016). Therefore, the oral cavity of a breastfed infant is constantly exposed to maternal bacteria, some of which may colonize the oral cavity (Boix-Amorós et al., 2016). Indeed, the human milk microbiome includes numerous taxa that are typical of the infant oral microbiome (Stinson et al., 2021). It is therefore hypothesized that breastfeeding shapes the oral microbiome via direct transfer of bacteria from milk to mouth. However, given the phenomenon of retrograde flow of milk from the infant oral cavity back into the mammary gland, it is difficult to determine the directionality of this strain sharing (oral to milk or milk to oral). To disentangle this relationship, one study of 17 mother-infant pairs examined gestational mammary secretions (week 38–40) and matched infant oral samples taken at 5–7 days after delivery from exclusively breastfed infants using bacterial culture and whole genome sequencing (Ruiz et al., 2019). Interestingly, most mother-infant pairs were found to have shared taxa between gestational milk secretions and oral samples that were almost indistinguishable, with a similarity of >99.9% (Ruiz et al., 2019). This finding supports the hypothesis human milk bacteria seed the oral microbiome. In another study of nine mother-infant pairs, the infant oral microbiome across the first six months of life showed a high degree of similarity with paired milk, mammary areola, and maternal oral samples (Drell et al., 2017). In a similar study of 36 exclusively breastfed infants many of the dominant infant oral taxa were found to be shared with paired human milk samples (Biagi et al., 2017). However, these two studies relied on short-amplicon sequencing, which provides poor taxonomic resolution. Strain-level studies are needed to better assess the degree of bacterial transmission between human milk and the infant oral microbiome. Milk-oral strain sharing should be considered in light of the likely bidirectional nature of this relationship (Biagi et al., 2018; Ruiz et al., 2019).

#### 4.2.2. Human milk oligosaccharides

In addition to microbes, non-microbial components in human milk such as HMOs, potentially influence the infant oral microbiome. HMOs are a group of minimally digestible, structurally complex glycans that represent the third most abundant solid component of human milk (Doare et al., 2018). To date, approximately 200 different structures have been identified (Ninonuevo et al., 2006). HMO composition varies between mothers and is based on maternal genetics (Andreas et al., 2015; Hegar et al., 2019). The average concentration of HMOs in human milk varies from 20 to 24 g/L in colostrum to 10–15 g/L in mature milk (Andreas et al., 2015; Thurl et al., 2017; Xu et al., 2017; Zhang et al., 2021).

In addition to acting as decoy receptors for potentially pathogenic bacteria and viruses, HMOs also have prebiotic actions that promote the growth of certain bacterial taxa, particularly Bifidobacterium species (Marcobal and Sonnenburg, 2012). Little is known about the role of HMOs in the development of the oral microbiome. Only one recent study (*n* = 10) examined the association between daily intakes of HMOs and the composition of the exclusively breastfed infant oral microbiome (Cheema et al., 2022). This study reported numerous relationships between oral taxa and infant HMO intakes across the first four months of life, which varied over time and were dependent on maternal secretor status (Cheema et al., 2022; Table 1). One interesting finding from this study was a negative correlation between intake of 3′-fucosyllactose (3’FL) and lacto-N-fucopentaose II (LNFP II) and infant oral *S. epidermidis*. Given that *S. epidermidis* strains have demonstrated virulence and resistance mechanisms that may implicated them in adult dental caries, HMOs may set children up for oral health later in life (Devang Divakar et al., 2017). Additionally, an *in vitro* study examined the impact of 2′-fucosyllactose (2’FL) and galacto-oligosaccharides (GOS) on *S. mutans* (a pathogen of dental caries), finding that 2′FL and GOS have a protective effect against *S. mutans* by inhibiting its adhesion to and growth on saliva-coated hydroxyapatite (Salli et al., 2020). These findings highlight the potential protective effect of HMOs within the infant oral microbiome. Nevertheless, due to the dearth of research on HMOs and their association with early-life oral microbiome, their functions within this environment are still largely unexplored. Therefore, large cohort studies together with *in vitro* experiments are required to explore the function of HMOs in the infant oral microbiome.

**Table 1:** Summary of associations between HMOs and oral bacteria.

Author	HMOs	Oral bacteria	Association
Cheema et al. (2022)	LNnT	*Haemophilus haemolyticus* *Bifidobacterium longum* subsp. *infantis*	Positive
	LNFP I	*Bifidobacterium longum* subsp. *infantis* *Haemophilus parainfluenzae* *Veillonella* sp. oral clone ASCB03	Positive
	LNFP II	*Staphylococcus epidermidis* *Bergeyella* sp.	Negative
	LNFP III	*Haemophilus parainfluenzae* *Veillonella* sp. oral clone ASCB03	Positive
	LSTb	*Veillonella nakazawae*	Positive
	LSTc	*Gemella haemolysans*	Negative
	LNH	*Bifidobacterium longum* subsp. Infantis *Veillonella* sp. oral clone ASCB03	Positive
	FLNH	*Streptococcus salivarius*	Positive
	3FL	*Bifidobacterium longum* subsp. infantis *Staphylococcus epidermidis* *Veillonella* sp. oral clone ASCB03	Negative
	DFLac	*Haemophilus parainfluenzae* *Streptococcus mitis*	Positive
	DFLNT 3′SL	*Veillonella nakazawae*	Positive
		*Haemophilus parainfluenzae* *Streptococcus mitis*	Positive
	6′SL	*Bifidobacterium longum* subsp. Infantis *Veillonella* sp. oral clone ASCB03	Positive
		*Rothia mucilaginosa* *Streptococcus mitis*	Negative
	LNT	*Streptococcus salivarius*	Negative
	DSLNH	*Bifidobacterium longum* subsp. infantis	Positive
		*Rothia mucilaginosa* *Streptococcus mitis*	Negative
	DSLNT	*Haemophilus haemolyticus* *Haemophilus parainfluenzae* *Veillonella* sp. oral clone ASCB03 *Veillonella nakazawae*	Positive
		*Gemella haemolysans* *Streptococcus mitis* *Veillonella* sp. oral clone ASCB03	Negative
	2′FL	*Bifidobacterium longum* subsp. Infantis *Veillonella* sp. oral clone ASCB03	Positive
	FDSLNH	*Streptococcus salivarius*	Positive
Salli et al. (2020)	2′ FL	*Streptococcus mutans*	Negative
	GOS	*Streptococcus mutans*	Negative

LNnT, lacto-N-neotetraose; LNFP, lacto-N-fucopentaose; LNFP II, lacto-N-fucopentaose II; LNFP III, lacto-N-fucopentaose; LSTb, sialyl-lacto-N-tetraose b; LSTc, sialyl-lacto-N-tetraose c; LNH, lacto-N-hexaose; FLNH, fucosyllacto-Nhexaose; 3FL, 3-fucosyllactose; DFLac, difucosyllactose; DFLNT, difucosyllacto-N-tetrose; 3′SL, 3′-sialyllactose; 6′SL, 6′-sialyllactose; LNT, lacto-N-tetrose; DSLNH, disialyllactoN-hexaose; DSLNT, disialyllacto-N-tetraose; 2′FL, 2′-fucosyllactose; FDSLNH, fucodisialyllacto-N-hexaose; GOS, galacto-oligosaccharides.

#### 4.2.3. Short-chain fatty acids

Short-chain fatty acids (SCFAs) are microbial metabolites produced by bacterial fermentation of dietary fiber in the colon (Koh et al., 2016). SCFAs have multiple impacts on host health, including providing energy for coloncytes, regulation of glucose homeostasis (Zadeh-Tahmasebi et al., 2016), regulation of lipolysis and adipogenesis (Lee and Hossner, 2002), anti-tumor effects (He et al., 2021), and anti-inflammatory activities (Huuskonen et al., 2004). Human milk contains detectable levels of the SCFAs acetate, butyrate, and formate (Stinson et al., 2020). SCFAs are produced by the maternal gut microbiota and can pass from maternal gut into the mammary glands via the circulation (Stinson et al., 2020). SCFAs act as substrates for bacterial metabolism and may thereby shape the infant oral microbiome by promoting the growth of SCFA-metabolizing taxa.

Nevertheless, SCFAs, in particular butyrate, may have a potential detrimental effect on the oral epithelial cells by inducing epithelial detachment and cell death. The production of SCFAs by periodontal pathogens can impact immune and periodontal cells, which hinders the host’s ability to defend against pathogenic bacteria (Magrin et al., 2020). To date, no studies have investigated the role of SCFAs in human milk on the oral microbiome.

#### 4.2.4. Human milk antimicrobial proteins

Human milk contains three major antimicrobial proteins: lactoferrin, lysozyme, and secretory immunoglobulin A (sIgA). Lactoferrin is an iron-binding glycoprotein synthesized in the mammary glands. The highest concentration of human milk lactoferrin is reported in colostrum, with levels decreasing gradually during the first month of life (Rai et al., 2014). Lactoferrin is an important host defense molecule that possesses anti-bacterial, anti-viral, and anti-fungal properties. Lactoferrin sequesters environmental iron, limiting the availability of this resource for bacterial growth (Jenssen and Hancock, 2009). Lactoferrin also has direct bactericidal effects via destruction of the bacterial cell membrane of Gram-negative bacteria (Ellison et al., 1988; Jenssen and Hancock, 2009). The ability of lactoferrin to kill *S. mutans* via an iron-independent mechanism has been reported (Arnold et al., 1981) and indeed therapeutic use of lactoferrin has been shown to be effective against an number of different oral pathologies such as gingivitis, periodontitis, halitosis, xerostomia, and alveolar or maxillary bone damage (Rosa et al., 2021) While lactoferrin is also present in saliva (Rosa et al., 2021), the concentration of lactoferrin in milk is far higher (Yang et al., 2018; Czosnykowska-Łukacka et al., 2019), suggesting that milk is a significant source of lactoferrin for the breastfed infant oral cavity. Thus, human milk lactoferrin may alter the infant oral microbiome by reducing potential pathogenic colonization.

Lysozyme is an enzyme that has the ability to kill bacteria by hydrolyzing the glycosidic bonds in the bacterial cell membrane of Gram-positive bacteria which represent the majority of the infant oral bacteria (Mason et al., 2018; Ferraboschi et al., 2021). In contrast, Gram-negative bacteria are resistant to lysozyme because their outer cell membrane consists of lipopolysaccharide, which restricts lysozyme diffusion. Indeed, lysozyme is present in infant saliva at similar levels to those seen in adults (Tenovuo et al., 1986). Salivary lysozyme influences the oral bacteria and contributes to oral health (Tenovuo et al., 1987; Kobus et al., 2017). An *in vitro* study examined the bactericidal properties of lysozyme on oral bacteria (Iacono et al., 1980). Inhibition of *S. mutans* growth was observed for more than 20 h after exposure to lysozyme (Iacono et al., 1980). Additionally, lysozyme is more abundant in colostrum (1–5 days after birth) than in mature milk (Montagne et al., 2001). Human milk lysozyme may influence early oral colonization dynamics by restricting the ability of potential pathogens to colonize. Evidence from pig models suggests that lysozyme-rich milk enhances beneficial microbes (*Bifidobacteriaceae* and *Lactobacillaceae*) and reduces detrimental microbes within the gut (Maga et al., 2012). However, its function within the infant oral cavity is currently unexplored.

sIgA is the predominant immunoglobulin present in human milk (Dingess et al., 2021). It is produced by plasma cells located within the mucous membranes lining the intestine and transported to the mammary glands via the circulation (Corthesy, 2013; Pietrzak et al., 2020). sIgA is more concentrated in colostrum than mature milk (Czosnykowska-Łukacka et al., 2020), with concentrations decreasing gradually over the first 12 weeks postpartum (Kawano and Emori, 2013). The major functions of sIgA are inhibition of bacterial adhesion to mucosal surfaces and neutralization of viruses by inhibition of transcytosis (Liu and Newburg, 2013). While no studies have directly examined the impact of human milk sIgA on the infant oral microbiome, it may be hypothesized that sIgA would prevent adhesion of some milk bacteria to the infant oral cavity. Indeed, approximately 40% of milk bacteria are sIgA coated (Dzidic et al., 2020) and higher levels of salivary sIgA have been related to a reduction in dental caries in children (Soesilawati et al., 2019). Thus sIgA may have a protective function for oral health.

As the breastfed infant mouth is constantly exposed to human milk during feeding, concentrations of antimicrobial proteins in human milk are likely important for oral microbial development. In fact, given that these antimicrobial proteins are broken down by the infant digestive tract (Elwakiel et al., 2020), there is likely a greater influence of intact proteins on the oral microbiome than on the gut microbiome. Therefore, further research is required to elucidate the relationship between antimicrobial proteins in human milk and the oral microbiome during the breastfeeding period.

### 4.3. Duration of breastfeeding

Weaning is a critical transition point in infancy associated with growth and developmental changes (Nuzzi et al., 2022). It is well-known that cessation of breastfeeding drives maturation of the infant gut microbiome (Bäckhed et al., 2015), however, no such studies have been performed for the oral microbiome. Interestingly, in a study of the oral microbiome of donkey foals, significant changes were observed during the weaning period compared with pre-weaning period (Zhang Z. et al., 2022). While a direct comparison of pre-and post-weaning is yet to be performed in humans, there is some data to suggest that the duration of breastfeeding may impact the oral microbiome.

The effect of breastfeeding duration on the composition of the oral microbiome has been studied in a cohort of 90 children at 3, 6, 12, and 24 months and 7 years of age (Dzidic et al., 2018). Interestingly, at 24 months of age, infants who remained partially breastfed until at least 12 months of age differed significantly in their oral microbiomes compared to those who were no longer receiving human milk by 12 months of age (Dzidic et al., 2018). This effect was long lasting, with a distinct difference in the communities still detected at 7 years of age. Among children who breastfed until at least 12 months of age, a higher relative abundance of Streptococcus at 12 months and Veillonella at 7 years was observed. Among those who ceased breastfeeding before 12 months of age, a higher relative abundance of Porphyromonas at 12 months, Neisseria at 24 months, and Actinomyces at 7 years was observed (Dzidic et al., 2018). Porphyromonas is involved in periodontitis, which destroys gingiva (gums) and can lead to tooth loss (Mysak et al., 2014). This implies that early cessation of breastfeeding may put infants at risk of later-life oral diseases. However, given that cessation of breastfeeding prior to 12 months requires supplementation with infant formula, it is difficult to differentiate the effects of lack of breast milk *per se* from the effects of formula use. Further, the cariogenic effect of infant formula has been evaluated in 36 children aged 1–2 years, who were fed with infant formula milk three times a day for 21 days (Chaudhary et al., 2011). A significant increase in the colony-forming units of *Streptococcus mutans*, a caries associated species, was observed in the oral microbiome of supplemented infants (Chaudhary et al., 2011). Thus, the impact of infant formula on the oral microbiome and oral health should be evaluated in further research.

Evidence on the impact of breastfeeding duration on the oral microbiome is sparse. This is a significant gap in the evidence, due to the controversy surrounding the association between prolonged breastfeeding and dental caries (Li et al., 2000; Rosenblatt and Zarzar, 2004; Vachirarojpisan et al., 2004; Azevedo et al., 2005; van Palenstein Helderman et al., 2006; Yonezu et al., 2006; Avila et al., 2015; Tham et al., 2015; Cui et al., 2017; Peres et al., 2017; Chanpum et al., 2020; van Meijeren-van Lunteren et al., 2021; Suparattanapong et al., 2022). Some studies have shown a protective effect of breastfeeding against tooth decay compared to exclusively formula feeding (Avila et al., 2015; Cui et al., 2017) While others have shown that breastfeeding beyond 9 months increases the risk for tooth decay (Li et al., 2000; Rosenblatt and Zarzar, 2004; Vachirarojpisan et al., 2004; van Palenstein Helderman et al., 2006; Tham et al., 2015; Cui et al., 2017; Peres et al., 2017; Chanpum et al., 2020; van Meijeren-van Lunteren et al., 2021). This issue must be resolved in order to provide evidence-based recommendations to parents. Thus, breastfeeding duration and exclusivity should be considered in future studies to better understand the long-term consequences of breastfeeding duration on the oral microbial composition and oral health.

### 4.4. Feeding preterm infants

Human milk, often with fortification, is the nutrition recommended for preterm infants (Gu et al., 2021). However, mothers of preterm infants may be unable to produce sufficient volumes of milk to meet their infant’s needs, necessitating the use of alternative nutrition, such as donor human milk or formula. Further, preterm infants are often administered milk via nasogastric tubes, bypassing exposure to the oral cavity. As such, oral administration of colostrum has become standardized in many NICUs due to its ability to enhance the weight gain, decrease the duration of hospitalization, and decrease the risk of poor health outcomes (Huo et al., 2022). In a small study of 20 preterm neonates, the effect of oropharyngeal colostrum administration on the oral microbiome was investigated (Cortez et al., 2021). The study found a higher relative abundance of oral Staphylococcus, Bifidobacterium, and Bacteroides in the intervention group compared with those receiving standard care (Cortez et al., 2021). Similar findings, including increased oral Staphylococcus, were reported in another study of 12 very low birth weight infants (Sohn et al., 2016). Another consideration for preterm infants is the use of fortified human milk. While the impact of milk fortification on the gut microbiome has been studied (Underwood et al., 2013; Cong et al., 2017; Dahl et al., 2018; Cai et al., 2019), there is an absence of data about the effects on the oral microbiome. Such differences in neonatal nutrition may have consequences on the oral microbiome and long-term oral health.

Preterm birth is known to influence dental development and be associated with dental caries (Seow, 1997; Schüler et al., 2018). Delayed growth and development of both primary and permanent dentition has also been observed in preterm children (Seow, 1997). Children born preterm are also at a significantly higher risk for dental caries (50% prevalence in preterm children compared to 12.5% in term children) (Schüler et al., 2018). However, early feeding modes have not been investigated in these studies. The effect of preterm infant feeding practices on the composition of the oral microbiome should be examined in future cohorts to better support oral health in the preterm population.

### 4.5. Introduction of solids

In the first year of life, infants progress through their diet, from milk (either human milk or infant formula) to solid food. The WHO recommends that solid food is introduced at six months of age [World Health Organization (WHO), 2021b]. Introduction of solid foods not only introduces new substrates for bacterial metabolism, but also results in a gradual reduction in the dose of human milk, which may also lead to changes in the composition of the oral microbiome (Oba et al., 2020). However, limited studies have investigated the impact of the introduction of solid food on the oral microbiome (Sulyanto et al., 2019; Oba et al., 2020).

The dramatic change in nutrient sources that accompanies the introduction of solids is likely to influence the diversity and composition of the oral microbiome (Dzidic et al., 2018). In one study of 12 infants, oral samples collected immediately pre-and post-introduction of solid foods were compared. On average, solid food was introduced to the infant diet at 6.8 ± 0.9 months of age, following which the infant oral microbiome became richer and more diverse, and the relative abundance of *Streptococcus mitis* was reduced (Sulyanto et al., 2019). In another study of 12 infants sampled at birth and 2, 4, and 6 months of age, samples taken after the introduction of solids (*n* = 1 at 4 months, *n* = 10 at 6 months) had a high abundance of Gemella, Neisseria, Veillonella, and Fusobacterium compared to samples taken prior to the introduction of solids (Oba et al., 2020). While shifts in the oral microbiome have been detected around the time of introduction of solids, it is important to consider that introduction of solid food occurs close to the time of primary tooth eruption, which may contribute to the acquisition of new species by providing new surfaces for bacterial attachment (discussed further below) (Wan et al., 2001).

Overall, solid food introduction may increase the complexity of the infant oral microbiome. Due to limited evidence on the impact of solid food introduction on the oral microbiome, large longitudinal studies with detailed feeding data are needed. Such studies may aid in separating the effects of solid food introduction and tooth eruption events.

## 5. Non-dietary factors that shape the infant oral microbiome

### 5.1. Tooth eruption

Tooth eruption may also impact the microbial composition of the oral cavity. Teeth begin to erupt in the infant oral cavity at approximately six months of age with a full complement of primary teeth at around 30 months of age, providing new surfaces for bacterial adhesion. As tooth emergence and the introduction of solid food often occur at the same time (Kageyama et al., 2019; Welch et al., 2020), it is difficult to differentiate between the effects of these two events.

The complexity of the oral microbiome increases with the emergence of primary teeth (Xu et al., 2022). In a study of 21 infants, saliva and plaque samples were taken across five detention states: prior to tooth eruption (~4–5 months old; saliva only), following the eruption of the first two teeth (~6 months old; saliva and plaque), following the eruption of the upper and lower primary incisors (~9 months old; saliva and plaque), following the eruption of the first molars (~14–19 months old; saliva and plaque), and at full dentition (~24–36 months old; saliva and plaque) (Xu et al., 2022). Approximately 30% of the OTUs in saliva and 70% of those in plaque remained across all dentition states, suggesting that the majority of saliva taxa are altered by tooth eruption, while the majority of plaque taxa remain. Streptococcus dominated the saliva across all dentition states, but declined gradually with teeth eruption, accompanied by a rise in community diversity. Following primary tooth eruption, new taxa colonize the oral cavity, including species of Streptococcus, Neisseria, Rothia, Veillonella, Corynebacterium, Gemella, Treponema, and Granulicatella (Shi et al., 2016; Mason et al., 2018; Xu et al., 2022).

Further maturation of the oral microbiome in childhood may be associated with the transition from primary to permanent dentition (Shi et al., 2018). There is a large overlap in taxa present prior to and following permanent dentition, with Actinomyces strongly associated with permanent teeth (Shi et al., 2016; Mason et al., 2018; Shi et al., 2018). These findings indicate that the oral microbiome at primary tooth eruption has a significantly higher alpha diversity compared to pre-dentate infants, mixed, and permanent dentations (Mason et al., 2018). Although dentition is a crucial milestone in infant oral microbiome development, few studies have specifically characterized the impact of tooth eruption on the oral microbiome. Further work is required to clarify whether the development of the oral microbiome is primarily influenced by age or by the emergence of teeth, with a focus on various sites within the oral cavity which may be differentially impacted by the emergence of teeth (Xiao et al., 2020).

### 5.2. Dental caries

Dental caries is the most prevalent childhood oral disease globally. It is estimated that 514 million children have primary teeth caries (Yu et al., 2021). Dental caries is also associated with alterations to the oral microbiome (Zhang J. S. et al., 2022). Factors such as consumption of sugar, frequency of breastfeeding, and night-time breastfeeding, have been impact the oral microbiome potentially increasing the incidence of dental caries (Carrillo-Diaz et al., 2021).

The etiology of dental caries occurs in three main steps. Firstly, biofilm is formed on the surface of teeth via formation of conditioning film, which allow cell-to-surface attachment of the primary colonizers and cell-to-cell interaction of the late colonizers. Then, teeth are coated with salivary components such as glycoproteins, mucins, sialic acid, and bacterial cells debris, which are absorbed on to the teeth enamel. After that, primary colonizer bacteria, such as *Actinomyces viscosus* and *Streptococcus sanguis,* interact with the biofilm by several cell-to-surface pathways (Lamont et al., 1991; Davey and O’toole, 2000). This interaction is influenced by carbon source, pH, and osmolarity (Davey and O’toole, 2000). The third step is adhesion of pathogenic bacteria such as *S. mutans* to the initial colonizers, leading to dental caries (Davey and O’toole, 2000). Although dental caries may be caused by many microorganisms (Aas et al., 2008; Holgerson et al., 2015; Hurley et al., 2019a), *S. mutans* has been identified as a common causative agent (Tanner et al., 2011; Holgerson et al., 2015; Hurley et al., 2019a). *Streptococcus mutans* is able to process monosaccharides to acid and to produce extracellular polysaccharides, leading to caries (Shellis and Dibdin, 1988; Zero, 2004).

The relationship between the oral microbiome and caries is likely bidirectional: the oral microbiome can cause caries, and caries can impact the oral microbiome. The oral microbiome of caries-active children is reduced in alpha diversity and Veillonella, and enriched in *Streptococcus mutans* and other streptococci, Cutibacterium, Prevotella, and Lactobacillus (Belstrøm et al., 2014; Richards et al., 2017; Wang et al., 2017; Xu et al., 2018; Wang et al., 2019; Hurley et al., 2019a; Yang et al., 2021).

While dental caries alter the oral microbiome, differences in the infant oral microbiota may precede childhood caries. The ability of the infant oral microbiome to predict later childhood caries has been investigated prospectively (Xu et al., 2018; Dashper et al., 2019;Grier et al., 2021; Blostein et al., 2022). In a recent case–control study, the oral microbiome prior to the onset of caries was depleted in Neisseria, *Haemophilus parainfluenzae*, and *Fusobacterium periodonticum* compared to controls (Blostein et al., 2022). This shift in the microbiome was also associated with a decrease in saliva pH and an increased risk of acquiring *S. mutans*. Importantly, this study controlled for numerous potential confounders, including maternal education, primary dentition, mode of delivery, breastfeeding, and antibiotic exposure (Blostein et al., 2022). In a similar prospective study of 134 children, 65 of whom developed caries by age of 5 years, the oral microbiome was characterized at seven time points from 1.9 months to 5 years of age (Dashper et al., 2019). While children who would go on to develop caries and those who would remain healthy could not be discriminated based on their oral microbiota at prior to 20 months of age, distinctions began to develop by 39 months of age. A number of taxa were identified as discriminatory between children who would go on to develop caries and those who would remain healthy; however, presence of *S. mutans* was the largest predictor of later disease. Similarly, in a study of 56 children 1–3 year old children who were sampled 6-monthly, 36 of which went on to develop caries, machine learning models were able to predict the onset of early childhood caries with an AUC of 0.71 at 1 year of age, and an AUC of 0.89 in the visit immediately before disease onset, suggesting a progression in the oral microbiome toward disease (Grier et al., 2021). In these models, *Rothia mucilaginosa*, *Streptococcus* sp., and *Veillonella parvula* were the most discriminatory species. While alpha diversity is known to be reduced in children with caries (Belstrøm et al., 2014; Hurley et al., 2019a), alpha diversity has not been reported to be predictive of caries (Grier et al., 2021; Blostein et al., 2022). This suggests that the reduction in alpha diversity that occurs in caried-affect children occurs as a result of the disease. Together, a shift in composition of the oral microbiome of children who developed caries compared to those who remained healthy suggests a role for the infant oral microbiome in dental caries susceptibility. If such shifts can be identified early, timely intervention may be applied to promote oral health in children.

### 5.3. Respiratory diseases

Respiratory bacteria may also contribute to the infant oral microbiome. Anatomically, the oral cavity is also a route for the respiratory tract. Mutual exchange of microbes between the respiratory tract and the oral cavity may occur due to mouth breathing and coughing (Dong et al., 2022). Typical taxa of the respiratory system, such as Haemophilus and Pseudomonas, are regularly identified in the infant oral cavity (Segal et al., 2013).

Several studies in adults have shown an association between oral bacteria and the risk of respiratory diseases such as influenza, pneumonia, and chronic obstructive pulmonary disease (Scannapieco and Ho, 2001; Pragman et al., 2019; Takeuchi et al., 2019; Gomes-Filho et al., 2020; Imai et al., 2021). This finding indicates that respiratory health may be associated with the oral microbiome. However, this association has not been examined in infants. Therefore, studies of the infant microbiome should include data on respiratory health to clarify this link.

### 5.4. Antibiotic exposure

Many infections are treated by antibiotics in early life, which have systemic effects regardless of the site of infection. Antibiotic use is widespread in infants, with 82.3% of children prescribed antibiotics within the first two years of life in New Zealand (Leong et al., 2020). Exposure to antibiotics therefore has the potential to disrupt the oral microbiome (Lazarevic et al., 2013; Dzidic et al., 2018; Kennedy et al., 2019).

Studies have reported an association between antibiotic use in early life and perturbations to the oral microbiome (Lazarevic et al., 2013; Dzidic et al., 2018; Kennedy et al., 2019). However, the long-term effects of this are not fully understood. Recently, the impact of antibiotic intake in the first two years of life on the oral microbiota was assessed in Swedish children (*n* = 90), who were followed from birth to 7 years of age (Dzidic et al., 2018). Of those, 30% had been treated with amoxicillin or phenoxymethylpenicillin in their first year of life, while 44% had been treated with those antibiotics in their second year of life. Interestingly, the oral cavities of unexposed children exhibited a higher relative abundance of *Neisseria* spp., *S. mitis, and Streptococcus dentisani-species* associated with childhood caries (Gross et al., 2010). Thus, antibiotics exposure may influence early oral health. However, to date, no association has been reported between early-life antibiotic use and dental caries. Similar results were reported from another cohort in which the use of antibiotics was categorized according to “ever-used” and “used within the last three months” at the time of sample collection. Antibiotics were prescribed for 6, 3, and 16% of children at ages 6, 12 and 24 months, respectively. Exposure to antibiotics within the three months prior to sample collection was positively associated with *Pasteurellaceae* and *Neisseriaceae*, and negatively associated with *Prevotellaceae* (Kennedy et al., 2019). Corresponding results were reported from a smaller study of 18 children aged 12–72 months treated with amoxicillin and a control group (*n =* 15) who were not exposed to antibiotics. Saliva samples were collected at the initial visit (at the prescription of amoxicillin), approximately 10 days later (at the end of treatment), and a month after commencement of antibiotics (approximately 3 weeks after cessation of treatment). Samples were collected from the control group at the same three time points. Exposure to amoxicillin was associated with a major shift in the relative abundances of various taxa and a reduction in species diversity and richness in the oral cavity. At the end of the treatment period, a reduction in Streptococcus, Gemella, Rothia, Fusobacterium, and Prevotella was observed in the treated group. Notably, the oral microbiome exhibited partial recovery three weeks after the course of amoxicillin but remained distinct from pre-antibiotic samples in terms of richness and diversity (Lazarevic et al., 2013). These findings together imply a significant and lasting impact of antibiotics on the early oral microbiome.

### 5.5. Other factors

Aside from the factors discussed here, there are various potential factors that may influence the early-life oral microbiome. A small number of studies have shown an association between oral microbiome composition in early life and maternal delivery mode (Li et al., 2005; Holgerson et al., 2011; Nelun Barfod et al., 2011; Thakur et al., 2012; Dominguez-Bello et al., 2016; Li H. et al., 2018; Hurley et al., 2019b), intrapartum antibiotic prophylaxis (Gomez-Arango et al., 2017; Li et al., 2019), gestational diabetes mellitus (He et al., 2019), infant pacifier use (Plonka et al., 2012), and ethnicity (Premaraj et al., 2020; Table 2). However, study numbers and consistency between studies is low. Additionally, there are other early-life factors and events which may influence the development oral microbiome, such as second-hand smoke exposure, teething interventions (gels, toys, washcloths), and teeth brushing behavior (cloth, silicon or plastic bristle, brushing frequency, age of introduction to toothpaste). However, to date, these have not been explored in infants.

**Table 2:** Studies examining determinants of the oral microbiome in early life.

Factor	Study	Location	Sample size	Age at sampling	Sample type	Method	Major finding
Delivery mode	Li H. et al. (2018)	China	18 CSD 74 VD	Immediately post-partum	Oral buccal swab	16S rRNA gene sequencing V3-V4	VD associated with higher abundances of Lactobacillus, Bifidobacterium, Corynebacterium, Bacteroides, and Ureaplasma. CSD associated with higher abundances of Petrimonas, Bacteroides, Desulfovibrio, Pseudomonas, Staphylococcus, Tepidmicrobium, VadinCA02, and Bifidobacterium.
	Hurley et al. (2019b)	Ireland	84	<1 wk.,1, 2, 6, 12 mo	Oral buccal swab	16S rRNA gene sequencing V4-V5	At 1 wk. CSD infants had a higher abundance of Streptococcus and Gemella. At 1 wk. VD infants had a higher abundance of Porphyromonas and Prevotella. No effect on the infant oral microbiome beyond 1 mo of age.
	Thakur et al. (2012)	India	30 CSD 30 VD	3, 6, 9, 12 mo	Oral buccal swab and plaque swab	Bacterial culture	No correlation.
	Holgerson et al. (2011)	Sweden	38 CSD 25 VD	3 mo	Oral buccal swab	16S rRNA sequencing	31 species were detected only in VD infants. 6 species were detected only in CSD infants.
	Nelun Barfod et al. (2011)	Sweden	42 CSD 42 VD	6–10 mo	Oral buccal swab	DNA–DNA hybridization	VD associated with higher relative abundances of *Streptococcus salivarius, Lactobacillus salivarius, Lactobacuillus casei, Lactobacillus curvata.*
	Dominguez-Bello et al. (2010)	Venezuela	6 CSD 4 VD	< 5 min after delivery	Oral buccal swab	16S rRNA gene sequencing V2	VD associated with higher Lactobacillus, Prevotella.
Intrapartum antibiotic prophylaxis	Gomez-Arango et al. (2017)	Australia	36	≤ 3 d	Oral buccal swab	16S rRNA gene sequencing V6-V8	Higher abundance of Proteobacteria in exposed neonates. *Streptococcaceae, Gemellaceae* and *Lactobacillales* dominated in unexposed neonates.
	Li et al. (2019)	China	22 infants Exposed = 11 Unexposed = 11	Immediately post-partum	Oral buccal swab	16S rRNA gene sequencing V3-V4	Higher abundance of *Lactobacillus, Bacteroides, Bifidobacterium,* and *Corynebacterium* in exposed neonates.
Gestational diabetes mellitus	He et al. (2019)	China	Exposed = 9 Unexposed = 11	Immediately post-partum	Oral buccal swab	16S rRNA gene sequencing V3-V4	GDM associated with lower abundance of Firmicutes and higher alpha diversity, Streptococcus, and Faecalibacterium.
Pacifier use	Plonka et al. (2012)	Australia	214	6, 12, 18, 24 mo	Oral buccal swab and plaque swab	Bacterial culture	At 24 mo, *S. mutans* colonization was significantly associated with addition of condiments to pacifier.
Ethnicity	Premaraj et al. (2020)	US	96 African American = 26 Burmese = 19 Caucasian = 26 Hispanic = 25	6–11 yrs. (sampled once)	Plaque swab	16S rRNA gene sequencing V3-V4	Most complex supragingival microbiome in Burmese infants. Supragingival microbiome varied among ethnicity groups.

CSD, caesarean section delivered; VD, vaginally delivered; GDM, gestational diabetes mellitus; min, minutes; d, day; wk, week; mo, month; yrs, year.

## 6. Horizontal and vertical transmission of oral microbiota

Sources and transmission routes for oral microbiota in early life remain under explored. Recent data suggests that there is a high level of horizontal transmission of oral strains among household members, with a median of 32% of strains shared between co-habituating individuals (Valles-Colomer et al., 2023). Parents are also a major source of vertical transmission of oral strains to their infants, with recent data demonstrating a higher degree of strain sharing between mothers and infants than between fathers and infants (Valles-Colomer et al., 2023). This higher level of transmission from mother persists into adulthood, with mothers sharing more OTUs with their adult children than fathers (Sundström et al., 2020). Strain sharing between mothers and infants begins early, with a 16.4% transmission rate within the first three days of life (Ferretti et al., 2018). The high degree of strain sharing between mothers and infants has been attributed to maternal behaviors such as spoon sharing, oral pacifier cleaning, and kissing (Virtanen et al., 2015; Damle et al., 2016), as well as breastfeeding (Biagi et al., 2017; Drell et al., 2017; Ruiz et al., 2019). Maternal and horizontal sources of the infant oral microbiome have recently been reviewed elsewhere, with the authors of this review additionally identifying childcare and school settings as a potential source of strain sharing (Azevedo et al., 2023).

Given that mothers are major donors of bacterial strains to the developing infant oral microbiome, factors that influence the maternal microbiome, such as maternal infection, antibiotic exposure, maternal smoking, or maternal immune status, may also alter the infant oral microbiome (Retnakumari and Cyriac, 2012; Gomez-Arango et al., 2017; Goto et al., 2019; Li et al., 2019; Coker et al., 2020; Sobiech et al., 2023). For example, in a study of HIV exposed and unexposed children, maternal immune status was found to be more significantly associated with her child’s oral microbiome than the child’s exposure to HIV, with subsequent implications for risk of caries (Coker et al., 2020). It is therefore necessary to consider the infant oral microbiome in the broader context of maternal, infant, and household health.

## 7. Summary and future directions

In this review, we have summarized current evidence on the development of the oral microbiome in early life, with a focus on the impact of infant feeding practices. Limited studies have investigated the influence of the human milk and other factors on the infant oral microbiome. Due to the dynamic nature of early microbiome assembly, future longitudinal studies should include large sample sizes and extend through infancy and childhood. In particular, detailed dietary, teething, and health data should be collected to better understand relationships with the oral microbiome in early life. Importantly, given significant differences in the health impacts of various Streptococcus species, it is important to use techniques such as full-length 16S rRNA gene sequencing to obtain species level resolution. Additionally, this review highlighted a significant gap regarding the potential influence of human milk antimicrobial proteins on the oral microbiome. Future research should measure concentrations and intakes of human milk antimicrobial proteins to assess their impact on the oral microbiome. Understanding the assembly of the oral microbiome will facilitate the development of strategies to support infant and lifelong oral and systemic health.

## Author contributions

RA: original draft preparation. LS and DG: conceptualization, and review and editing. LS, DG, and CL: supervision. All authors have read and agreed to the published version of the manuscript.

## Funding

RA, CL, DG, and LS are supported by an unrestricted research grant from Medela AG, administrated by the University of Western Australia. Ministry of Education, Saudi Arabia provides a PhD scholarship for RA.

## Conflict of interest

The authors declare that the research was conducted in the absence of any commercial or financial relationships that could be construed as a potential conflict of interest.

## Publisher’s note

All claims expressed in this article are solely those of the authors and do not necessarily represent those of their affiliated organizations, or those of the publisher, the editors and the reviewers. Any product that may be evaluated in this article, or claim that may be made by its manufacturer, is not guaranteed or endorsed by the publisher.
